# An Integrated Analysis of miRNA and Gene Expression Changes in Response to an Obesogenic Diet to Explore the Impact of Transgenerational Supplementation with Omega 3 Fatty Acids

**DOI:** 10.3390/nu12123864

**Published:** 2020-12-17

**Authors:** Karla Fabiola Corral-Jara, Laura Cantini, Nathalie Poupin, Tao Ye, Jean Paul Rigaudière, Sarah De Saint Vincent, Alexandre Pinel, Béatrice Morio, Frédéric Capel

**Affiliations:** 1 Unité de Nutrition Humaine (UNH), Université Clermont Auvergne, Institut National de Recherche pour L’agriculture, L’alimentation et L’environnement (INRAE), Faculté de Médecine, F-63000 Clermont-Ferrand, France; karla-fabiola.corral-jara@inrae.fr (K.F.C.-J.); jean-paul.rigaudiere@inrae.fr (J.P.R.); sarah.de_saint_vincent@uca.fr (S.D.S.V.); alexandre.pinel@uca.fr (A.P.); 2 Computational Systems Biology Team, Institut de Biologie de l’Ecole Normale Supérieure, CNRS, INSERM, Ecole Normale Supérieure, Université PSL, 75005 Paris, France; cantini@bio.ens.psl.eu; 3 Toxalim (Research Centre in Food Toxicology), Université de Toulouse, INRAE, ENVT, INP-Purpan, UPS, 31027 Toulouse, France; nathalie.poupin@inrae.fr; 4 GenomEast Platform, Institut de Génétique et de Biologie Moléculaire et Cellulaire (IGBMC), 1 rue Laurent Fries/BP 10142/, 67404 Illkirch, France; yetao@igbmc.fr; 5 CarMeN Laboratory, INSERM U1060, INRAE U1397, Université Lyon 1, 69310 Pierre Bénite, France; beatrice.morio@inrae.fr

**Keywords:** metabolic syndrome, transcriptome, microRNA, liver, omega 3 fatty acids, obesity

## Abstract

Insulin resistance decreases the ability of insulin to inhibit hepatic gluconeogenesis, a key step in the development of metabolic syndrome. Metabolic alterations, fat accumulation, and fibrosis in the liver are closely related and contribute to the progression of comorbidities, such as hypertension, type 2 diabetes, or cancer. Omega 3 (*n*-3) polyunsaturated fatty acids, such as eicosapentaenoic acid (EPA), were identified as potent positive regulators of insulin sensitivity in vitro and in animal models. In the current study, we explored the effects of a transgenerational supplementation with EPA in mice exposed to an obesogenic diet on the regulation of microRNAs (miRNAs) and gene expression in the liver using high-throughput techniques. We implemented a comprehensive molecular systems biology approach, combining statistical tools, such as MicroRNA Master Regulator Analysis pipeline and Boolean modeling to integrate these biochemical processes. We demonstrated that EPA mediated molecular adaptations, leading to the inhibition of miR-34a-5p, a negative regulator of Irs2 as a master regulatory event leading to the inhibition of gluconeogenesis by insulin during the fasting–feeding transition. Omics data integration provided greater biological insight and a better understanding of the relationships between biological variables. Such an approach may be useful for deriving innovative data-driven hypotheses and for the discovery of molecular–biochemical mechanistic links.

## 1. Introduction

Weight gain and abnormal lipid metabolism are major determinants of whole body insulin resistance, leading to the appearance of cardiovascular diseases, type 2 diabetes, cancer, etc. [1]. Insulin resistance corresponds to the decreased ability of insulin to stimulate glucose uptake in muscle, adipose, and other tissue, and to inhibit hepatic gluconeogenesis. Under normal conditions, a fasted state is characterized because glucagon can activate hepatic gluconeogenesis, lipolysis, and the oxidation of fatty acids (FA). Upon feeding, insulin released from pancreatic B cells reaches hepatocytes to inhibit gluconeogenesis and promote glycolysis and de novo lipogenesis [2]. With insulin resistance, these mechanisms are dysregulated, as no more glucose enters the cell, gluconeogenesis and lipolysis are increased. Inhibition of insulin signaling may arise from the deposition of FA or other lipid mediators in the peripheral tissues, proteins secreted by dysfunctional/inflamed adipose tissue through intracellular mechanism which remain partially characterized [3]. Several reports have suggested that metabolic abnormalities and alterations of adipose tissue biology could be prevented by increasing the intake of omega-3 (*n*-3) polyunsaturated fatty acids (PUFAs), especially those of marine origin such as eicosapentaenoic acid (EPA) or docosahexaenoic acid (DHA) [4,5,6].

Promising and higher effects of EPA compared to DHA were described against obesity and insulin resistance, but the molecular mechanisms leading to a differential effect are not yet fully clarified and the long term or transgenerational effects are unknown [7]. Hence, metabolic adaptations during pregnancy may be influenced by the lipid quality of the diet, inducing a differential response to a nutritional stress, probably linked to epigenetic modifications [8]. The integration of multi-omic data can provide deeper biological insights, a better understanding of relationships between biometric, biological, molecular variables. Innovative data-driven hypotheses paving the way to the discovery of mechanistic links could then be proposed. Recently, an integration of multi-omic data allowed the study of calorie restriction-induced changes in insulin sensitivity and the identification of diverse biomarkers [9]. On the other hand, the analysis of large data sets generated by metabolomics and lipidomics has shed new light on the roles of metabolites, such as lipids, amino acids and bile acids, in modulating insulin sensitivity. Metabolites can regulate insulin sensitivity directly by modulating components of the insulin signaling pathway, such as insulin receptor substrates (Irs) and AKT, and indirectly by altering the flux of substrates through multiple metabolic pathways, including lipogenesis, lipid oxidation, protein synthesis and degradation and hepatic gluconeogenesis [10].

In this study, we used a systems biology approach to explore hepatic metabolic and regulatory networks in response to an obesogenic diet in mice supplemented with EPA during three generations. We extracted cues of the underlying molecular mechanisms from transcriptomic and miRomic high-throughput data, using statistical methods, such as MicroRNA Master Regulator Analysis (MMRA) pipeline, and Boolean modelling. We were able to predict microRNA (miRNA)–gene interactions and simulate hepatic metabolic pathways of glucose flux in silico. From this analysis, we were able to extract new regulations between Irs2 gene and miR-34a-5p linked to transgenerational intake of EPA as a master controller of hepatic gluconeogenesis to prevent insulin resistance

## 2. Materials and Methods

### 2.1. Dietary Intervention and Sample Collection

The present study was approved by the Animal Care and Use Committee of Auvergne (CEMEA Auvergne) and the Ministère de l’Enseignement Supérieur et de la Recherche (01276.01). C57bl/6J mice were bred for 3 generations in the animal facility the INRAE research center of Theix (63122 Saint-Genes Champanelle, France) with a growing diet (A03 diet from Safe diets, Augy, France) supplemented with 1% (*w*/*w*) of fish oil (Polaris Omegavie 70 TG, EPA) containing 75% of omega 3 FA (omega 3 lineage), mainly in the form of EPA which represented 8% of the fatty acids in the final diet or 1% (*w*/*w*) of high oleic sunflower oil (control lineage) containing 83.5% of oleic acid (Lesieur, Coudekerque Branche, France) as a control group. The supplementation of the control group with 1% of sunflower oil aimed at matched the caloric supplementation related to the addition of fish oil in the omega 3 lineage with a marginal impact on metabolic parameters. The details of the breeding strategy and lipid composition of the diets are described in Supplementary Figure S1. F3 male mice from control and omega 3 lineages were matched for body weight (*n* = 8 per group) and fed with a high-fat, high-sucrose diet (HFD, 24% of fat, 20% of sucrose) providing 45% of energy from fat (RD 12,451 from Research diet, Brogaarden Gentofte, Denmark) for 17 weeks. These animals constituted the HFoleic and HFepa groups, respectively. A reference group of F3 male mice (*n* = 9) from the control lineage was fed with a low-fat diet (LFD) providing 10% of energy from fat (RD 12450H from Research diet) during the challenge as a reference group. Animals were maintained under a temperature-controlled environment and 12 h–12 h light-dark cycle throughout the study. All the procedures were followed to reduce the number and manipulation of the animals in the study.

At the end of the feeding period, during the fasting period, the animals were sacrificed under anesthesia with 4% isoflurane. Liver was harvested, snap-frozen in liquid nitrogen and stored at −80 °C until use. Total RNA was extracted from the liver using TRIzol^®^ reagent (Thermo Scientific, Courtaboeuf, France) according to the manufacturer’s instructions. Each total RNA sample was assessed for quantification and integrity using the Agilent Bioanalyzer (Agilent, Santa Clara, CA, USA).

### 2.2. Gene Expression and miRNA Analysis

#### 2.2.1. Microarray Gene Expression Analysis

Gene expression profiles were performed at the GeT-TRiX facility (INRAE, GénoToul, Génopole Toulouse Midi-Pyrénées, Toulouse, France) using Agilent Sureprint G3 Mouse GE v2 microarrays (8 × 60 K, design 074809) following the manufacturer’s instructions. For each sample, 200 ng of total RNA were prepared for hybridization using the One-Color Quick Amp Labeling kit (Agilent Technologies, Les Ulis, France) following the manufacturer’s instruction. All samples had an RNA integrity number above 7.5. The slides were scanned on Agilent G2505C Microarray Scanner (Agilent Technologies, Les Ulis, France) using Agilent Scan Control A.8.5.1 software (Agilent Technologies, Les Ulis, France) and fluorescence signal extracted using Agilent Feature Extraction software v10.10.1.1 (Agilent Technologies, Les Ulis, France). Raw data (median signal intensity) were filtered; log2 transformed and normalized using quantile method [11]. A model was fitted using the limma lmFit function [12]. Microarray data and experimental details are available in NCBI’s Gene Expression Omnibus [13] and are accessible through GEO Series accession number GSE145620 (https://www.ncbi.nlm.nih.gov/geo/query/acc.cgi?acc=GSE145620).

#### 2.2.2. Small RNA Sequencing (Small RNA-Seq) Analysis

Small RNA-seq were performed at the IGBMC GenomEast Platform (Strasbourg, France), small RNA-seq libraries were generated from 2000 ng of total RNA using TruSeq Small RNA Library Prep Kit (Illumina, San Diego, CA, USA), according to manufacturer’s instructions. Briefly, during the first step, RNA adapters were sequentially ligated to each end of the RNA, firstly the 3′ RNA adapter (5′-TGGAATTCTCGGGTGCCAAGG-3′) which is specifically designed to target miRNAs and other small RNAs, then the 5′ RNA adapter (5′-GTTCAGAGTTCTACAGTCCGACGATC-3′). Small RNA ligated with 3′ and 5′ adapters were reverse transcribed, and PCR amplified (30 s at 98 °C; (10 s at 98 °C, 30 s at 60 °C, 15 s at 72 °C) × 13 cycles; 10 min at 72 °C) to obtain cDNA. Acrylamide gel purifications of 140–160 nt amplified cDNA (corresponding to cDNA obtained from small RNA +120 nt from adapters) were performed. The final cDNA libraries were checked for quality and quantified using capillary electrophoresis. Libraries were loaded in the flow cell at 2.8 nM and clusters were generated using Cbot and sequenced on HiSeq 4000 (Illumina) as single-end 50 base reads, according to manufacturer’s instructions. Adapters were removed using FASTX-Toolkit (http://hannonlab.cshl.edu/fastx_toolkit/index.html). The trimmed sequencing reads of each library were then mapped and annotated with ncPRO-seq [14] onto the mus musculus genome GRCm38.p6 genome (mm9). Briefly, read alignment was performed using Bowtie v0.12.8, allowing a sum of qualities of mismatching bases lower than 50 (denoted –e 50). Aligned reads were then annotated accordingly against ortholog miRNAs in mice (miRBase release 22.1). MiRNA data were Log2 transformed and normalized using the quantile method [11]. MiRNA data and experimental details are described in GEO accession GSE146445 (https://www.ncbi.nlm.nih.gov/geo/query/acc.cgi?acc=GSE146445).

### 2.3. Bioinformatic and Statistical Analysis

#### 2.3.1. Gene Differential Expression between Biological Conditions

Pair-wise comparisons between biological conditions (reference, HFoleic, HFepa) were performed using T-test and Fold Change (FC). A correction for multiple testing was applied using the Benjamini–Hochberg procedure [15] to control the false discovery rate (FDR). Genes with FDR-adjusted *p* < 0.05 were considered to be differentially expressed between conditions. For the combination of gene and miRNA expression data (see Section 2.3.3), we defined as “up signature genes”, those with log2 FC > 0 and FDR-adjusted *p* < 0.05, and as “down signature genes” those with log2 FC < 0 and FDR-adjusted *p* < 0.05 in each biological comparison (HFepa vs. reference, HFoleic vs. reference and HFepa vs. HFoleic).

#### 2.3.2. Testing Gene Signature Performances with Partial Least-Squares Discriminant Analysis and Hierarchical Clustering

In order to verify the classification group (condition) of our samples, we performed a partial least squares regression-discriminant analysis (PLS-DA). PLS-DA was performed from differentially expressed genes and miRNA using Mixomics R [16]. In addition, hierarchical clustering of samples and gene expression data was performed using Euclidean distance and Ward’s method.

#### 2.3.3. Application of MicroRNA Master Regulator Analysis (MMRA)

The four steps of the MicroRNA Master Regulator Analysis (MMRA) pipeline [17] was applied to identify miRNA-gene interactions possibly involved in the differential biological regulations in each comparison (HFepa vs. reference and HFoleic vs. reference). MMRA consists of four sequential steps, each aimed at progressively reducing the number of candidate miRNAs: (i) differential expression analysis to highlight miRNAs with subtype-specific expression; (ii) target gene transcript enrichment analysis, to further select those miRNAs whose predicted targets are enriched in the associated subtype gene signature; (iii) network analysis, in which an mRNA network is constructed around each miRNA and tested for enrichment in signature genes; and (iv) identification of miRNAs whose expression “explains” the expression of subtype signature genes.

- MMRA step1: MiRNA differential expression analysis

Pair-wise comparisons between biological conditions in miRNA expression data were performed using the Bioconductor package DEGseq [18] to capture the differentially expressed miRNAs. miRNAs with FC > 0.25, *p* < 0.01 (corresponding to an FDR < 0.05) [15] were defined as differentially expressed between biological conditions and retained for further analysis.

- MMRA step2: target genes enrichment analysis

For each miRNA differentially expressed in a biological condition, miRNA target genes were predicted by combining the results of three miRNA-target databases (miRTarBase [19], miRDB [20], and TargetScan [21]). Only miRNA-target interactions predicted by at least two databases were included as putative targets in our analysis. We performed a target enrichment analysis in the “up” and “down” gene signature of the biological condition in which the miRNA was found differentially expressed. The enrichment analysis was performed with a Fisher’s exact test. The miRNAs whose targets were enriched with a Fisher’s *p* < 0.05 were retained for the following steps of the pipeline.

- MMRA step3: network analysis

ARACNE information-theoretic algorithm (http://wiki.c2b2.columbia.edu/califanolab/index.php/Software/ARACNE) was used to infer interactions between each miRNA selected in the previous step and any gene from the paired dataset. The software was available through the Cyni Toolbox panel under the Infer Network tab, implemented in Cytoscape (https://cytoscape.org/). Quantile normalization was performed as standard ARACNE pre-processing. Only edges connecting miRNA to genes were considered, thus having the miRNA as the only hub of the network. A mutual information (MI) *p*-value significance threshold fixed at 10^−7^ was used as recommended. Each of the consensus networks was tested for significant enrichment in “up” and “down” gene signature of each biological condition with Fisher’s exact test. A threshold of *p* < 0.05 was used in the enrichment test.

- MMRA step4: Step-wise Linear Regression (SLR) analysis

SLR is here used to filter out weak miRNA-gene interactions identified in the previous steps. In the SLR analysis, the gene log2-expression levels were considered as response variables, and the log2-expression levels of miRNAs linked by ARACNE or by databases to the gene were considered as the explanatory variables. Akaike information criterion was used as the stop criterion. At the end of this step, for each biological condition, we obtained a list of miRNAs with their associated signature genes.

### 2.4. Pathways Analysis

In order to gain mechanistic insight into gene lists generated from our previous analysis, we performed a pathway enrichment analysis, and we identified related biological pathways.

#### 2.4.1. Molecular Pathway Analysis with GeneTrail2

Molecular signatures from HFepa vs. reference, HFoleic vs. reference and HFepa vs. HFoleic comparisons were explored using gene set enrichment analysis (GSEA) implemented in GeneTrail2 [22,23]. In order to limit the effect of outliers, the unweighted version of GSEA implemented in GeneTrail2 evaluated whether the genes of different biological categories from KEGG-, Reactome- and Wiki-pathway datasets were randomly distributed or accumulated on top or bottom of the list. To this aim, a Kolmogorov–Smirnov test was applied on FC of genes with a differential expression between 2 groups at *p* < 0.05 with no FDR adjustment. As recommended by the GeneTrail2 algorithm, only sets of genes identified at *p* < 0.05 after adjustment for multiple testing using the Benjamini–Yekutieli method were considered.

#### 2.4.2. Metabolic Pathways Analysis with MetExplore

The list of genes exhibiting a differential expression and regulated by at least one miRNA was used for visualization and exploration of metabolic pathways using MetExplore [24] (https://metexplore.toulouse.inra.fr/metexplore2/). This list of genes was mapped on the mouse metabolic network iSS1393 [25] (biosource Id #2893 in MetExplore) to find the associated metabolic reactions. The identified reactions were assigned a different numerical attribute if they were associated with differentially expressed genes in the HFoleic condition (1), HFepa condition (2) or in both conditions (3) compared to reference group. The whole list of reactions with their numerical attributes was then mapped on the metabolic network to locate them in the whole metabolic network and visualize associated metabolic pathways of interest. Pathway enrichment analyses were performed over the sets of reactions associated with the HFoleic condition, the HFepa condition and common to both condition, respectively, to assess whether the given reactions were significantly over-represented in a metabolic pathway. Pathway enrichment statistics were performed using one-tailed exact Fisher test, with a Benjamini–Hochberg correction for multiple tests, using the metabolic pathways defined in the iSS1393 network.

### 2.5. Logical Modeling of Gene Regulatory and Metabolic Networks

A Boolean modelling approach implemented in GINsim software (v3.0.0b, http://ginsim.org) was used to build a dynamic model of the associations between the highlighted signaling pathways and the metabolic phenotypes related to obesity and insulin resistance [26]. This approach consisted in a simplified reconstruction of biochemical networks to connect metabolic components based on prior knowledge. The model inherently provided links of causality and an input–output relationship at the molecular level, allowing the in silico analysis of the dynamical behavior of the network (presence/activation, absence/deactivation of the different components) under different combinations of inputs or perturbations in the system [27].

Each node in the graph was represented by a single Boolean variable which exhibited two possible levels, 0 (OFF) if the element was absent/inactive or 1 (ON) if the element was present/active. For some exceptions, this two-state representation may be biologically too restrictive and 3 levels should be necessary, 0 (OFF), 1 (LOW), and 2 (HIGH), representing high or low activation of this node regulator in the network [28]. The model was represented on a regulatory graph where each node represented a component of the network. Edges represented the regulatory positive or negative influences between nodes. Next, we defined logical rules to determine the level of activity of each target node depending on the levels of regulators. Computational modeling through in silico simulations and perturbations revealed dynamical properties of the network. The model was then used to test and predict biological hypotheses. The model was built using input information from the literature, the KEGG database [29], and the present experiment.

### 2.6. Validation of Gene Expression

For microarray validation purposes, cDNAs were synthesized from 2 µg of total RNA using the High Capacity cDNA Reverse Transcription Kit from Applied Biosystem (Thermo Scientific, Courtaboeuf, France). The products of reverse transcription were used for Quantitative Real Time Polymerase Chain Reaction (qRT-PCR) using specific primers and Rotor-Gene SYBR Green PCR master mix on a Rotor-Gene Q system (Qiagen, Courtaboeuf, France). Quantification of mRNA of selected genes was assayed using the ddCT method using Hprt as housekeeping gene. Differential expression between reference, HFoleic and HFepa conditions were analyzed using pairwise T-test and a correction for multiple testing [15] using a cut-off for significance fixed at *p* < 0.05. Primer sequences and PCR conditions are available upon request (frederic.capel@inrae.fr).

## 3. Results

### 3.1. Effect of High Fat Diet and Transgenerational Supplementation with EPA in Gene and miRNAs Expression

The impact of the obesogenic diet on fat mass accumulation, increase in liver weight, plasma glucose, and insulin levels was reduced in mice that received a transgenerational supplementation with EPA as compared to the oleic/control lineage (Table 1).

**Figure 1:**
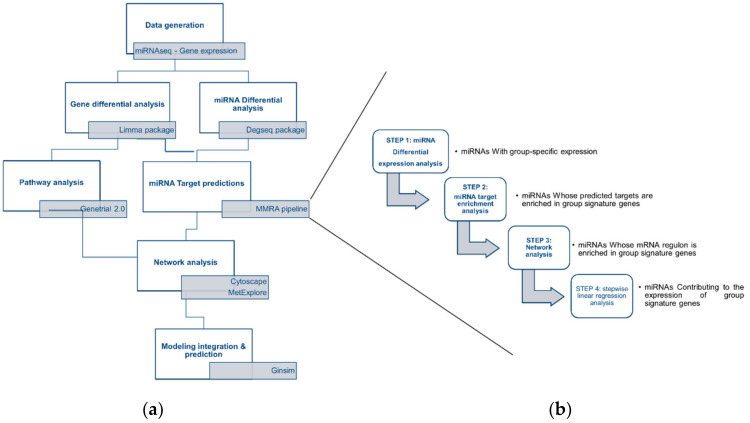
Bioinformatic workflow. (**a**) Bioinformatic analysis of microarray and microRNA-seq data was performed through a series of bioinformatic blocks: differential expression analysis, prediction of miRNA targets, analysis and visualization of interaction networks and computational modeling to predict cellular metabolic behaviors and phenotypes. (**b**) The four-step MMRA (microRNA Master Regulator Analysis) pipeline was followed to obtain the putative miRNA targets and included miRNA target enrichment analysis, network analysis and stepwise linear regression analysis.

The global workflow of our study was based on an integration of bioinformatic and statistical blocks, including the use of R packages, and software applications such as Cytoscape and GINsim (Figure 1). In order to go into the molecular mechanisms that could mediate the positive effect of supplementation with omega 3 FA in mice challenged with an obesogenic HFD; we characterized the adaptations at the level of miRNA and gene in the liver. We then analyzed differential expression between 3 biological conditions (HFepa, HFoleic and reference) as described in Section 2.3.1 and Section 2.3.3.

**Figure 2:**
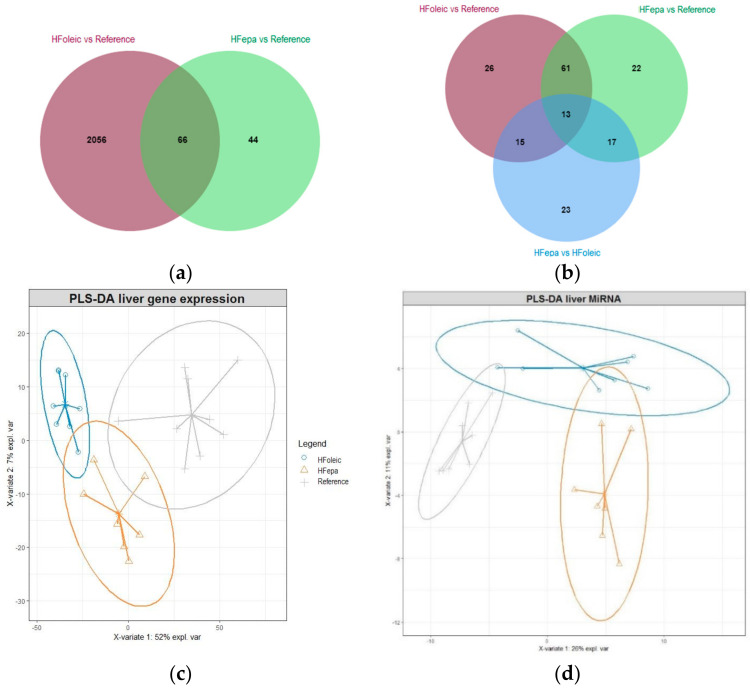
Effect of High Fat Diet and transgenerational supplementation with EPA in gene and miRNAs expression. (**a**) Venn diagram showing the relationships in the number of genes differentially expressed between HFepa vs. reference and HFoleic vs. reference biological condition. (**b**) Venn diagram showing the relationships in the number of miRNAs differentially expressed between three biological conditions, HFepa vs. reference, HFoleic vs. reference and HFepa vs. HFoleic. (**c**) Differentially expressed genes from three group biological condition, HFepa vs. reference, HFoleic vs. reference, HFepa vs. HFoleic were used to perform a partial least squares regression-discriminant analysis (PLS-DA). (**d**) PLS-DA analysis of differentially expressed miRNAs from three biological conditions, HFepa vs. reference, HFoleic vs. reference, HFepa vs. HFoleic.

As illustrated on the Venn diagrams showing the overlap of differentially expressed genes and miRNAs between comparisons (Figure 2a,b), a considerable difference in the number of genes regulated in HFepa and HFoleic groups, compared to the reference group was observed, whereas no significant differential expression of genes were detected between HFoleic and HFepa. Sixty-six genes were commonly regulated when HFepa and HFoleic were compared to reference group.

On the contrary, the number of differentially expressed miRNAs showed a balance between the biological conditions. Of these, 74 differentially expressed miRNAs were shared between HFepa and HFoleic conditions as compared to reference group. Thirty and twenty eight differentially expressed miRNAs were shared between HFepa vs. reference-HFepa vs. HFoleic comparisons and HFoleic vs. reference-HFepa vs. HFoleic comparisons respectively. The PLS-DA analysis showed a clear separation of the expression profiles in genes and miRNAs between groups (Figure 2c,d) but the expression profile of the HFepa group presented a similarity to the reference group.

### 3.2. MiRNAs Significantly Contribute to the Expression of Phenotype Signature Gene

The four-step sequential MMRA pipeline was applied to look over the regulation mediated by miRNAs on the differentially expressed genes. MMRA aims at progressively reduce the number of candidate miRNAs which could regulate one gene (Figure 1b). The four steps of the pipeline were differential expression analysis, target/transcript enrichment analysis, network analysis and Stepwise linear regression analysis as described in Section 2.3.3. As a result, miRNA-gene interactions were obtained with a strong statistical support. We performed a MMRA analysis for HFepa vs. reference and HFoleic vs. reference separately, showing, respectively, 12 and 32 potential miRNAs regulating the phenotype signature (Supplementary Tables S1 and S2). Table 2 shows down and up regulated target gene with the highest FC in the HFepa vs. reference comparison. Only seven of these genes were exclusively differentially expressed in the HFepa group compared to the Reference group. All the others were regulated after the obesogenic diet whatever the lineage. In the case of miRNAs, which could regulate the transcription of these genes, few of them were specific to each biological condition. Globally, downregulated genes are regulated by upregulated miRNAs and vice versa, although some exceptions were observed. Likewise, some differentially expressed genes in the HFepa group were regulated by miRNAs that were differentially expressed exclusively in the HFoleic group compared to the reference group.

### 3.3. Identification of Differentially Regulated Cellular and Signaling Pathways

**Figure 3:**
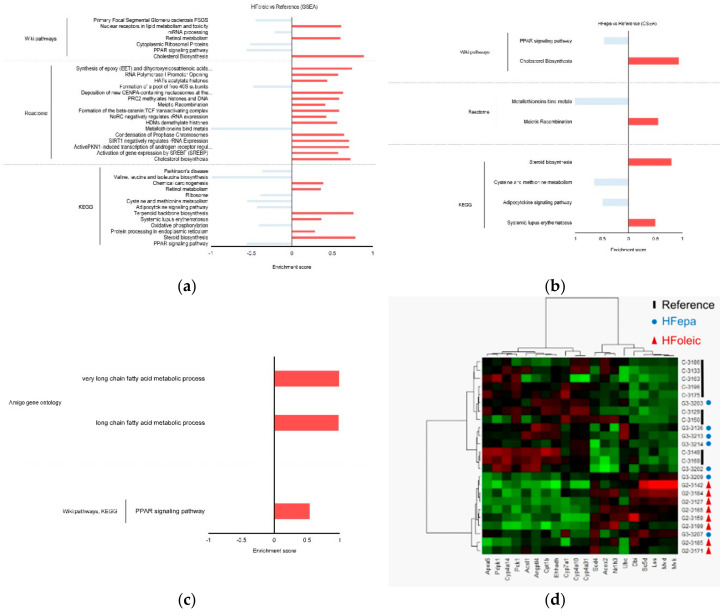
Gene set enrichment analysis of differentially expressed genes between conditions. (**a**) Histogram showing the enriched pathways in HFoleic condition vs. reference. (**b**) Histogram showing the enriched pathways in HFepa condition vs. reference. (**c**) Histogram showing the enriched pathways in HFepa condition vs. HFoleic condition. In panels (**a**)–(**c**), the red bars represent over representation of corresponding pathway or reaction; blue bars represent under representation of corresponding pathway or reaction. (**d**) Heat map visualization of expression data of the genes involved in peroxisome proliferator-activated receptor (PPAR) and Cholesterol pathways. The color code indicates the expression level of each genes and for each sample (green, low expression level; red, high expression level). The genes are represented as columns and the samples with the identification of group correspondence as rows.

Global pathway enrichment analysis on the three biological comparisons (HFoleic vs. reference, HFepa vs. reference and HFepa vs. HFoleic) using significant gene expression regulations (*p* < 0.05) with no FDR correction was performed with GSEA to relate genes and their biological functions. Enriched pathways were identified using the Wiki, Reactome, and KEGG databases. Pathways were mostly enriched in the HFoleic condition compared to the reference condition for which 8, 11 and 7 enriched pathways were found respectively (Figure 3a and Supplementary Table S3). Figure 3a exhibited a summary of this analysis, illustrating an enrichment of pathways related to oxidative phosphorylation, cholesterol metabolism, peroxisome proliferator-activated receptor (PPAR) signaling pathway, eicosanoid metabolism. Fewer pathways were enriched when HFepa and reference conditions were compared (Figure 3b, Supplementary Table S4). Enriched biological processes in this comparison were related to cholesterol metabolism, PPAR signaling pathway, sulfur-containing amino acid metabolism and adipocytokine signaling pathway. Comparison between the HFepa and HFoleic conditions showed a regulation of FA metabolism and PPAR signaling pathway (Figure 3c, Supplementary Table S5). Hierarchical clustering of expression data of genes related to PPAR and Cholesterol pathways identified in the HFepa vs. HFoleic biological conditions showed a well separation of samples according to their group, except for minor exceptions (Figure 3d).

### 3.4. Differential Enrichment of Metabolic Pathways in HFepa and HFoleic Groups

**Figure 4:**
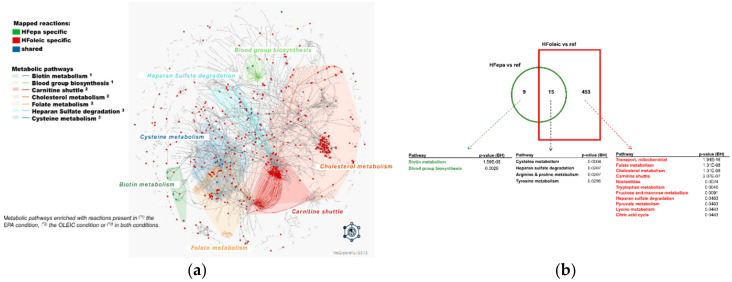
Connections between regulated metabolic pathways in HFoleic and HFepa goups. Genes differentially expressed from HFepa vs. reference and HFoleic vs. reference biological conditions were used to identify related metabolic reactions in MetExplore allowing a complementary exploration of enriched metabolic pathways. Metabolic reactions associated with modulated genes in both HFepa and HFoleic conditions were mapped in the Mus musculus metabolic network. (**a**) The Figure shows a global network of mapped metabolic reactions. Reactions in red are HFoleic-related; reactions in green are HFepa-related; blue reactions are both regulated in HFoleic and HFepa groups. Represented metabolic pathways correspond to metabolic pathways enriched with reactions present in the EPA condition ^(1)^, in the oleic condition ^(2)^ or in both conditions ^(3)^. (**b**) Representation of the number of identified reactions and the significantly enriched related metabolic pathways according to the MetExplore analysis in the two comparisons that exhibited significant differential gene expression (HFoleic vs. reference and HFepa vs. reference).

An identification of all potentially regulated enzymatic reactions using the MetExplore tool allowed a detailed extraction and visualization of metabolic sub-networks associated with differentially expressed genes in the HFepa and HFoleic groups compared to the reference condition (Figure 4a,b). As illustrated in Figure 4, all identified enriched metabolic pathways were represented as a network of reactions and metabolites (nodes) interactions (edges). Most of the reactions identified as enriched in the HFepa vs. Reference comparison were also enriched in the HFoleic vs. reference comparison (Figure 4b). The lists of metabolic pathways linked to all identified reactions and specifically enriched in each set of reactions from the different comparisons or shared between them can be found in Supplementary Table S6. Significant enrichment in the HFepa vs. Reference comparison concerned the metabolism of biotin and of blood group biosynthesis (Figure 4b). The most significantly enriched enzymatic reactions when HFoleic and reference groups were compared belonged to mitochondrial activity, fatty acid transport, and cholesterol. Enrichment in cysteine, arginine and proline metabolisms was shared between the two comparisons versus reference group. Carbohydrate metabolism (Glycolysis/gluconeogenesis) also strongly tended to be enriched (*p* = 0.06, Supplementary Table S6) and was identified to be connected to biotin and cysteine metabolisms (Supplementary Figure S2A and Figure S2B).

As illustrated in Supplementary Figure S2A,B, changes in one of the genes encoding the catalysts of a HFepa-specific reaction may be connected with reactions enriched in the HFoleic vs. reference-specific and vice versa. As an example, a connection between the biotin- (acetyl-CoA-carboxylase) ligase reaction (R_BACCL) and holocarboxylase synthetase’s reaction (R_BTNPL), both catalyzed by the HFepa-specific holocarboxylase synthetase (Hlcs) gene with the synthetase’s reaction (R_BTND1) catalyzed by the HFoleic-specific Biotinidase (Btd) gene was found (Supplementary Figure S2A). Interestingly, these reactions from biotin metabolism were connected by AMP with the glycolysis/gluconeogenesis and FA metabolism pathways (R_ACS, R_HEX1, R_PEPCK, R_ENO and R_ALDD2x). Likewise, the Glutamic-Oxaloacetic Transaminase 1 (Got1) gene, was differentially expressed in both HFepa and HFoleic groups compared to reference group. This enzyme is involved in L-Cysteate 2-oxoglutarate aminotransferase (R_LCYSTAT) and 3-sulfino-alanine transaminase (R_3SALATAi) reactions with 2-oxoglutanate as a common metabolite (Supplementary Figure S2B). These reactions from cysteine metabolism, could constitute a link between the changes in FA metabolism and glycolysis/gluconeogenesis through pyruvate and acetyl CoA in the control/oleic lineage.

### 3.5. MiRNAs Regulated Genes Involved in the Integration of Insulin Signaling, PPAR Signaling, Glucose Metabolism, and FA Metabolism

**Figure 5:**
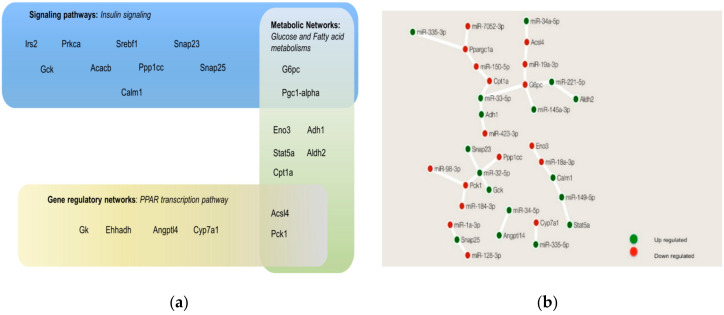
Interactions between miRNAs and genes in pathways related to insulin signaling, PPAR signaling, glucose and fatty acid metabolism. (**a**) Representation and compartmentalization of master genes involved in the selected pathways. (**b**) miRNAs-genes interactions of the genes represented in (**a**), obtained following the MMRA pipeline; green circles represented upregulated network components and red circles were downregulated network components. Construction of the network in (**b**) was done using Cytoscape software.

We explored the possible mechanisms linking miRNA and gene expression in the modulation of insulin resistance and the development of obesity using the MMRA pipeline. Based on the pathways identified in the GeneTrial2 analysis (see part 3) and the exploration of metabolic reactions (see part 4) as differentially affected between the three biological conditions, four cellular regulation layouts were selected. One signaling pathway: insulin signaling, one gene regulatory networks: PPAR signaling, and two metabolic networks: Glycolysis/Gluconeogenesis, and FA metabolism. These selected pathways were also highlighted in our GSEA analysis of the conditions between the HFepa and HFoleic groups (Supplementary Table S5). We extracted the list of genes that participated in each of the selected pathways and differentially expressed in our study and we identified the genes that were common in two or more of these pathways (Figure 5a). As a validation of this approach, the differential expression of several of these genes was confirmed by RT-qPCR (Supplementary Table S7). Subsequently, the mentioned genes with at least one miRNA regulation identified in the MMRA pipeline were presented in Figure 5b. This Figure shows a network of these recurrent genes and their regulation by miRNAs. As can be seen, Ppargc1a (or Pgc-1α), G6pc, Pck1, are genes that participate in two or more of the selected pathways, and could be regulated by two or more miRNAs, indicating that they are highly connected.

### 3.6. Computational Modeling Predicts that Dynamism in Genes and miRNAs Expression Leads to Specific Cell Metabolic Phenotypes

**Figure 6:**
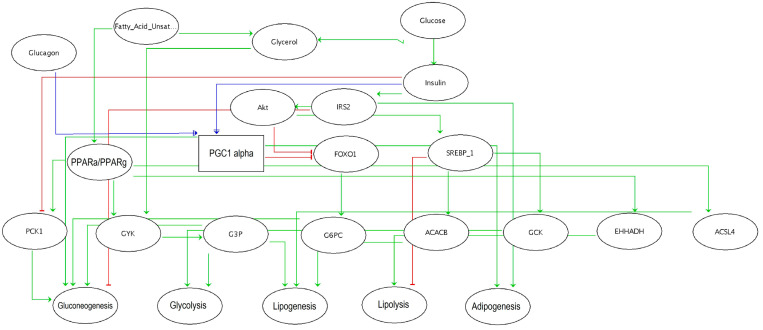
Computational modeling predicts that dynamism in genes expression leads to specific cell metabolic phenotypes. A Boolean modeling of glycolysis, gluconeogenesis, lipolysis, lipogenesis, and adipogenesis metabolic processes in the liver was obtained to create a logical regulatory graph of hepatic metabolism. The model encompassed 24 components (represented as ellipses) and 37 interactions (green edges = activation, red blunt = inhibition). A rectangle denoted a ternary component.

To achieve an integrated comprehension of our results and the dynamic of gene expression regulations involved in the mechanisms of insulin resistance and obesity, a logical bioinformatics model was built using the software GINsim (Figure 6). Our model included three inputs which could not be affected by other components of the network: glucagon, glucose, and FA; five outputs: gluconeogenesis, glycolysis, lipogenesis, lipolysis, and adipogenesis; and several internal elements like Irs2, Pck1, G6pc, Pgc-1α, Pparγ, and Pparα. The model was based on the observation that a high level of Pgc-1α expression during fasting shifts the ratio of Irs1:Irs2 toward a higher Irs2 expression to prime the insulin receptor response toward a more efficient inhibition of gluconeogenesis after feeding [30]. Pgc-1α was then considered with three possible activation levels (0,1,2) to simulate the regulation of metabolic pathways under diverse input conditions, such as the exposure to glucose during feeding. PPARs were introduced as activators of lipolysis, gluconeogenesis (through Ppar-α) and lipogenesis (through Ppar-γ), respectively.

**Figure 7:**
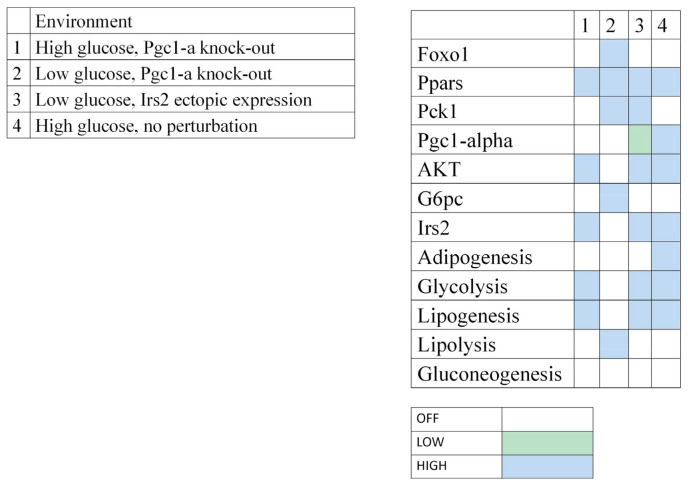
Model simulations in scenarios with perturbations. Context-dependent stable states computed for the model. Blank cell = inactivation of the corresponding component; blue cell = maximal activation (1 for Boolean components, 2 for Pgc-1α); green cell = intermediate level (1 for Pgc-1α only).

As calibration controls of the modeling and in agreement with prior knowledge, the presence of glucose lead to insulin, Irs2 and Akt activation, and consequently to glycolysis, lipogenesis, and adipogenesis (Figure 7, environment 4). On the contrary, when glucose was considered low, glucagon production was increased, while Akt and Irs2 remained inactivated, leading to the activation of lipolysis and gluconeogenesis (not shown).

Then, a series of in silico perturbations were performed to find a metabolic profile that could correspond to our experimental observations. Simulations were focused on Pgc-1α and Irs2 activation levels and compared to our experimental results. In the present study, we observed a stable expression of Pgc-1α and an upregulation of Irs2 expression in the HFepa group compared to the HFoleic condition in the context of a low level of circulating glucose (fasting state). Microarray data showed that both genes were downregulated in the HFoleic compared to reference mice. In the fasting state, simulation of a knockout of Pgc-1α (Figure 7, environment 2) resulted in the activation of lipolysis but not gluconeogenesis nor adipogenesis. Irs2 expression was null in this condition. In the context of environment 1, the simulation of a fed state (elevation of glucose in plasma), glycolysis and lipogenesis were still activated, even if Pgc-1α expression was absent (Figure 7). Finally, an ectopic expression of Irs2 was simulated when glucose level was low (Figure 7, environment 3), resulting in the inhibition of gluconeogenesis, but a maintained activation of glycolysis and lipogenesis. In this condition, Pgc-1α reached an intermediate expression value. This last condition probably best fitted to our experimental conditions.

No differential expression of Pgc-1α was observed between HFepa and HFoleic conditions. This may imply an adaptive mechanism to control glucose homeostasis independently of Pgc-1α after transgenerational supplementation with EPA and allowing an improvement of glucose homeostasis and insulin resistance. It raised the hypothesis that Irs2 protein was a master regulator of the differential molecular and biological adaptations between the two groups of mice receiving the obesogenic HFD diet. The MMRA pipeline was used to investigate how Irs2 gene could be regulated. It identified miR-34a-5p as a regulator of Irs2 expression. miR-34a-5p was downregulated in the HFepa condition compared to the HFoleic condition and upregulated in the HFoleic and HFepa conditions compared to the reference condition. Integrally, these data suggested that a new regulation between EPA-mediated miR-34a-5p-Irs2 and an inhibition of gluconeogenesis during fasting states through Irs2 overexpression could be clue mechanisms to modulate glucose flux and improve insulin resistance in an obesogenic environment.

## 4. Discussion

The omega-3 (*n*-3) long-chain polyunsaturated FAs (LC-PUFA), eicosapentaenoic (EPA, 20:5*n*-3) and docosahexaenoic (DHA, 22:6*n*-3) acids, are essential components of a healthy, balanced diet, having beneficial effects on development and in mitigating a range of pathological conditions [31]. LC-PUFAs control some key molecular cell mechanisms, resulting in a beneficial role in obesity and inflammatory diseases. Such mechanisms are complex and reflect the diversity of their functions, mainly as modulators of the dynamic properties of membranes, regulators of gene expression, and precursors of active mediators [32]. The development of obesity involved alterations in many biochemical processes and a pro-inflammatory state in insulin-sensitive tissues, leading to an increased risk of developing cardiovascular disease, type 2 diabetes, and cancer. The transgenerational modulation of the fatty acid profile of the diet was found to affect the susceptibility of the descendants [8] but the molecular adaptations remained to be elucidated. The liver has a key role in glucose and lipid homeostasis. Then, liver diseases are closely associated to these obesity-related morbidities. Transgenerational supplementation with EPA alleviated the metabolic impact of the HFD and limited fat mass accumulation in this early stage of weight gain. Beneficial effects were also observed against muscle metabolic disorders and energy expenditure [33]. In order to obtain further insights into the molecular mechanisms that contribute to the beneficial effects mediated by EPA, we integrated an analysis of hepatic gene and miRNA expression data using the statistically based MMRA pipeline. We implemented systems biology methodologies, such as Boolean modeling to simulate the metabolic processes of a cell under specific conditions. We were able to extract a new regulation between gene and miRNAs, which could play a key role in the inhibition of hepatic gluconeogenesis and the improvement of insulin resistance.

Transgenerational supplementation with EPA modulated hepatic gene expression and the related regulation by miRNAs in response to an obesogenic challenge in mice as compared to animals receiving a mixture of mono and saturated FA provided by a “high oleic” sunflower oil as a control condition. The diet supplemented with 1% of “high-oleic” sunflower oil was used to match the addition of energy provided by 1% of fish oil in the EPA lineage with a marginal metabolic impact. Most of the differentially expressed miRNAs and genes in the liver from mice exposed to a HFD were shared between the animals from the EPA and the control lineages. However, some of the genes were exclusive of the control/oleic or omega 3 lineage and could be involved in the differential metabolic response. Likewise, differentially expressed genes were analyzed to extract their miRNA regulation. Among the 30 mostly affected genes when HFepa and Reference groups were compared, some genes were regulated by miRNAs identified as differentially expressed in both HFepa or HFoleic groups, even some of these miRNAs were exclusive to the HFoleic lineage. Some genes had no known regulation by the identified miRNA and for which the mechanisms controlling their expression were not identified in our statistical analysis. Then, we performed a GeneTrial and MetExplore analysis to find the relations between our differentially expressed genes with general pathways or specific metabolic pathways, respectively. Our results provided new biological links between PPAR signaling, glucose metabolism, and FA metabolism as crucial determinants of the health effect of the transgenerational supplementation with EPA. We highlighted pathways related to amino acids, cholesterol, adipogenesis, biotin metabolism that converged on glucose and FA flux metabolism. Regulation of these pathways were related to pathologies mediated by obesity or metabolic syndrome. For instance, biotin is involved in gluconeogenesis, FA synthesis, amino acids catabolism by acting as a prosthetic group for pyruvate carboxylase, propionyl-CoA carboxylase, beta-methylcrotinyl-CoA carboxylase, and acetyl-CoA carboxylase [34]. On the other hand, oxoglutarate is at the intersection between carbon and nitrogen metabolic pathways [35]. Although experimental models should be developed to validate these links, our approach provided new potential targets for future therapeutic or preventive assays.

The integration of all our data suggested that the PPAR pathway appeared as a central axis in the differential adaptions to the obesogenic diet between the two lineages. The PPAR subfamily of nuclear receptors controls many different target genes involved in lipid metabolism, FA oxidation and glucose homeostasis [36,37]. PPARγ has a central role in adipogenesis and lipogenesis [38,39]. Heterozygous PPARγ-null mice exhibited greater insulin sensitivity than wild-type littermates and were protected from the development of insulin resistance and glucose intolerance mediated by a HFD [39]. As natural ligands for PPARs, could bind and activate PPARα to stimulate the expression of genes involved in FA oxidation and repress inflammation [40,41]. Omega-3 FA could also interact with PPARγ, but the impact of omega-3 FA binding to PPARγ on lipid metabolism remains controversial. An acute exposure to omega-3 FA had a stimulatory effect on PPARγ and adipogenesis, although a chronic exposure had a repressive effect [5]. Discrepancies between studies about the effect of EPA on PPAR transcription factors may have been linked to differences in the dose and time effect which should be considered to achieve a comprehension of modifications in insulin resistance and fat mass gain. Furthermore, it would be of scientific relevance to explore if similar adaptations occurred in the adipose tissue of these mice as PPARs are master regulators of adipose tissue biology.

Despite no differential expression of Pgc-1α gene between HFepa and HFoleic group, our results underscored the importance of Pgc-1α in glucose homeostasis in mice from the EPA lineage during the obesogenic diet. Pgc-1α is a member of a family of transcription coactivators which could interact with PPARγ in a ligand-independent manner. Pgc-1α was described to be highly abundant in tissues with high capacity for mitochondrial FA oxidation such as the brown adipose tissue, the heart, and skeletal muscle [42]. Hepatic Pgc-1α expression is induced in the liver during fasting and elevated during diabetes, causing an uncontrolled gluconeogenesis [43]. It was found that Pgc-1α promoted insulin resistance in the liver through a PPARα-dependent activation of the mammalian tribbles homolog TRB-3 [44], a fasting-inducible inhibitor of the serine-threonine kinase Akt/PKB [45]. A recent report showed that Pgc-1α had a crucial role in the control of gluconeogenesis during the fasting-to-fed transition [30] through the regulation of the balance between Irs1 and Irs2 expression, two major elements of the insulin-signaling pathway, during the fasting-to-fed transition to quickly modulate gluconeogenesis. Sensitization of the liver to insulin by Pgc-1α represented a necessary priming of the liver to shut down gluconeogenesis immediately after the rise of insulin during feeding [30], allowing a fine-tuning of plasma glucose level. Muscle-specific transgenic overexpression of Pgc-1α contributed to the development of diet-induced insulin resistance, probably due to an increase in FA and reduced glucose uptake, inducing intramuscular lipid accumulations and then the alteration of insulin signaling [46]. Altogether, these studies suggested that strong Pgc-1α overexpression, but not physiologic Pgc-1α overexpression, would induce harmful metabolic effects.

To help the interpretation of our observations, we used a model in silico of the cellular metabolic behavior under different scenarios by the integration of key variables or components which modulate glycolysis, gluconeogenesis, lipolysis, lipogenesis and adipogenesis processes as key modulator of metabolic homeostasis.

Our modeling analysis confirmed that an adequate expression level of Pgc-1α was required after feeding and that an intermediate level of Pgc-1α during the fasting state, resulted in the activation of lipolysis and gluconeogenesis. The simulation of Pgc-1α knockout resulted in the inhibition of both gluconeogenesis and adipogenesis, but an activation of lipolysis. This simulated state was likely to mimic the hepatic environment in mice from the omega 3 lineage during the obesogenic challenge as mechanisms to control glucose homeostasis and the accumulation of fat mass. In agreement with this, a recent report concluded that an inhibition of the FOXO1/Pgc-1α pathway regulated hepatic gluconeogenesis and improved insulin resistance in rats fed with a HFD and insulin-resistant cells [47].

As Pgc-1α gene level did not differ between control and EPA lineages, other factors, linked to the co-activator could modulate its activity. Our data and bioinformatics analysis suggested a contribution of insulin receptor substrate 2 (Irs2) in the effect of Pgc-1α against insulin resistance. Irs proteins are directly phosphorylated by the insulin receptor, leading to the recruitment and activation of additional signaling proteins [48]. Irs1 and Irs2 are crucial determinant of gluconeogenesis and lipogenesis in the liver and decreased hepatic Irs2 gene level was observed with hepatosteatosis [49,50,51]. These data strongly suggested that Irs2 is a key to achieve an understanding. Our data confirmed that Irs2 was a key element within the network of molecular mechanisms linked to insulin resistance. We showed differences in the levels of individual expression of the Irs2 gene between the HFepa vs. HFoleic conditions. Irs2 gene was upregulated in the HFepa group, while a downregulation of Irs2 expression was observed in the HFoleic group as compared to the reference group. Although it was proposed that Pgc-1α stimulated Irs2 expression [30], our data suggested that Irs2 could also be controlled by other mechanisms.

The expression of the Irs2 gene could be directly regulated by fixation of insulin, Srebp-1c and TF3 proteins to its promoter [52,53,54]. A negative control by miRNAs was also demonstrated in pancreatic beta cells [55]. A crucial role for miRNAs was described in the control of the integration of metabolism and mitochondrial functions during the fed-fasted transitions [56] to prevent hepatic dysfunction and metabolic or aging-associated diseases.

We hypothesized that Irs2 was, at least partly regulated by miRNA-34a (miR-34a-5p). MiR-34a is a part of the p53 tumor suppressor network [57] and one of the major miRNAs involved in the production of insulin, pancreatic development and glucose homeostasis [58].

In agreement with the downregulation of miR-34a-5p in HFepa group compared to HFoleic group, the expression level of this miRNA was increased in the fatty liver in mouse models of obesity [59]. Increased circulating levels of miR-34a were also correlated with the severity of hepatic disease in patients with non-alcoholic fatty-liver disease (NAFLD) or type 2 diabetes [60,61].

## 5. Conclusions

Our integrative analysis allowed the extraction of information about the molecular events and gene–protein networks modulated by manipulating of the diet across generations and which could affect metabolic and physiologic susceptibility to nutritional stress. We concluded that mice from the EPA lineage exhibited an improved response against fat accumulation, insulin resistance through a regulatory axis involving Pgc-1α, Irs2, and miR-34a-5p. This hypothesis must now be validated using invalidation or overexpression models targeting these proteins.

## Figures and Tables

**Table 1:** Metabolic characteristics of mice after the dietary intervention.

Parameter-	Reference			HFoleic			HFepa		
Body Weight (g)	26.7	±	0.9	36.1	±	1 ***	33.8	±	2.7 **
Fat %	16	±	1	32	±	1 ***	24	±	3 ** †
Lean %	79	±	1	64	±	1 ***	72	±	3 ** ††
Liver (gram)	0.87	±	0.04	1.11	±	0.04 *	0.99	±	0.09
AUCglucose (a.u.)	171	±	6	207	±	11 *	196	±	8
Glucose (mg/dL)	195.6	±	13.1	274.2	±	13.1 **	228.1	±	14.0 †
Insulin (pg/mL)	68.6	±	8.3	182.8	±	18.8 ***	109.4	±	25.9 †
TAG (g/L)	0.310	±	0.013	0.366	±	0.024	0.378	±	0.039
T Cholesterol (g/L)	0.926	±	0.034	1.116	±	0.050 *	1.166	±	0.068 *
Glycerol (µM)	213.1	±	11.9	237.2	±	19.0	257.1	±	24.0
Nefa (mM)	0.350	±	0.038	0.228	±	0.029	0.404	±	0.081

Data are mean ± SEM (*n* = 9, 8 and 7 mice in Ref, HFoleic and HFepa, respectively). * *p* < 0.05 vs. Ref; ** *p* < 0.01 vs. Ref; *** *p* < 0.001 vs. Ref; † *p* < 0.05 vs. HFoleic; †† *p* < 0.01 vs. HFoleic. TAG, triacylglycerols; Nefa, non-esterified fatty acids.

**Table 2:** Differentially expressed genes are regulated by miRNAs.

Gene	Associated Signature in HFepa	Associated Signature in HFoleic	miRNA Regulation	microRNA Regulation in HFepa	microRNA Regulation in HFoleic
*Sigmar1*	Down		mmu.miR.32.5p		up
*Xpa*	Down	Down	mmu.miR.150.5p		down
*Xpa*	Down	Down	mmu.miR.335.3p	up	up
*Xpa*	Down	Down	mmu.miR.1948.3p	up	up
*Rai1*	Down		mmu.miR.7052.3p		down
*Zfp777*	Down	Down	mmu.miR.335.3p	up	up
*Txn2*	Down	Down	mmu.miR.18a.3p		up
*Txn2*	Down	Down	mmu.miR.98.3p	down	
*Trpt1*	Down	Down	mmu.miR.148a.3p	up	
*Agpat6*	Down	Down	mmu.miR.195a.3p	down	down
*Leng9*	Down	Down	mmu.miR.7052.3p		down
*Plekhf1*	up	up	mmu.miR.150.5p		down
*Plekhf1*	up	up	mmu.miR.335.3p	up	up
*Stard4*	up	up	mmu.miR.32.5p		up
*Stard4*	up	up	mmu.miR.7052.3p		down
*Dnajb1*	up		mmu.miR.19a.3p		down
*Dnajb1*	up		mmu.miR.7068.3p	down	down
*Hspb1*	up	up	mmu.miR.128.3p	down	down
*Hspb1*	up	up	mmu.miR.150.5p		down
*Hspb1*	up	up	mmu.miR.7068.3p	down	down
*Pcp4l1*	up	up	mmu.miR.1948.3p	up	up
*A_55_P2525368*	Down		miRNA regulation not significant		
*Ptpmt1*	Down	Down	miRNA regulation not significant		
*Magohb*	Down		miRNA regulation not significant		
*A130022F02Rik*	Down	Down	miRNA regulation not significant		
*Gm13547*	Down	Down	no regulated by a miRNA		
*Lrrfip1*	Down	Down	no regulated by a miRNA		
*Rabl3*	Down	Down	no regulated by a miRNA		
*Kansl1l*	up	up	miRNA regulation not significant		
*P4ha1*	up		miRNA regulation not significant		
*Inhbb*	up		miRNA regulation not significant		
*Mtss1*	up	up	miRNA regulation not significant		
*Hyou1*	up	up	miRNA regulation not significant		
*Gbe1*	up	up	miRNA regulation not significant		
*Slc5a3*	up	up	miRNA regulation not significant		
*Ints6*	up	up	miRNA regulation not significant		
*0610031O16Rik*	up	up	no regulated by a miRNA		

Fifteen upregulated genes and 15 downregulated genes with the highest FC value (>0.25) in the HFepa condition were selected to demonstrate its regulation mediated by miRNAs. The four-step sequential MMRA pipeline was applied to obtain miRNA-gene regulations. The Table shows the regulatory pattern of genes and miRNAs in HFepa vs. Reference or HFoleic vs. Reference biological condition.

## Supplementary Materials

The following are available online at https://www.mdpi.com/2072-6643/12/12/3864/s1, Figure S1: Schematic representation of the dietary intervention, Figure S2: MetExplore pathways representation, Table S1: miRNAs regulating HFepa vs. reference phenotype signature (MMRA Analysis), Table S2: miRNAs regulating HFoleic vs. reference phenotype signature (MMRA Analysis), Table S3: Enriched pathways resulted from an overrepresentation analysis in GeneTrial 2.0. in HFoleic vs. reference, Table S4: Enriched pathways resulted from an overrepresentation analysis in GeneTrial 2.0. in HFepa vs. reference, Table S5: Enriched pathways resulted from a GSEA analysis in GeneTrial 2.0. in HFepa vs. HFoleic biological conditions, Table S6: Metabolic enriched pathways from MetExplore analysis in each group, Table S7: RT-qPCR validation of microarray data.

## References

[1] de Ferranti S., Mozaffarian D. (2008). The perfect storm: Obesity, adipocyte dysfunction, and metabolic consequences. Clin. Chem..

[2] Hatting M., Tavares C.D.J., Sharabi K., Rines A.K., Puigserver P. (2018). Insulin regulation of gluconeogenesis. Ann. N.Y. Acad. Sci..

[3] Samuel V.T., Shulman G.I. (2016). The pathogenesis of insulin resistance: Integrating signaling pathways and substrate flux. J. Clin. Investig..

[4] Albracht-Schulte K., Kalupahana N.S., Ramalingam L., Wang S., Rahman S.M., Robert-McComb J., Moustaid-Moussa N. (2018). Omega-3 fatty acids in obesity and metabolic syndrome: A mechanistic update. J. Nutr. Biochem..

[5] Martinez-Fernandez L., Laiglesia L.M., Huerta A.E., Martinez J.A., Moreno-Aliaga M.J. (2015). Omega-3 fatty acids and adipose tissue function in obesity and metabolic syndrome. Prostaglandins Other Lipid Mediat..

[6] Pinel A., Morio-Liondore B., Capel F. (2014). n-3 Polyunsaturated fatty acids modulate metabolism of insulin-sensitive tissues: Implication for the prevention of type 2 diabetes. J. Physiol. Biochem..

[7] Pinel A., Pitois E., Rigaudiere J.P., Jouve C., De Saint-Vincent S., Laillet B., Montaurier C., Huertas A., Morio B., Capel F. (2016). EPA prevents fat mass expansion and metabolic disturbances in mice fed with a Western diet. J. Lipid Res..

[8] Massiera F., Barbry P., Guesnet P., Joly A., Luquet S., Moreilhon-Brest C., Mohsen-Kanson T., Amri E.Z., Ailhaud G. (2010). A Western-like fat diet is sufficient to induce a gradual enhancement in fat mass over generations. J. Lipid Res..

[9] Dao M.C., Sokolovska N., Brazeilles R., Affeldt S., Pelloux V., Prifti E., Chilloux J., Verger E.O., Kayser B.D., Aron-Wisnewsky J. (2018). A Data Integration Multi-Omics Approach to Study Calorie Restriction-Induced Changes in Insulin Sensitivity. Front. Physiol..

[10] Yang Q., Vijayakumar A., Kahn B.B. (2018). Metabolites as regulators of insulin sensitivity and metabolism. Nat. Rev. Mol. Cell Biol..

[11] Bolstad B.M., Irizarry R.A., Astrand M., Speed T.P. (2003). A comparison of normalization methods for high density oligonucleotide array data based on variance and bias. Bioinformatics.

[12] Ritchie M.E., Phipson B., Wu D., Hu Y., Law C.W., Shi W., Smyth G.K. (2015). limma powers differential expression analyses for RNA-sequencing and microarray studies. Nucleic Acids Res..

[13] Edgar R., Domrachev M., Lash A.E. (2002). Gene Expression Omnibus: NCBI gene expression and hybridization array data repository. Nucleic Acids Res..

[14] Chen C.J., Servant N., Toedling J., Sarazin A., Marchais A., Duvernois-Berthet E., Cognat V., Colot V., Voinnet O., Heard E. (2012). ncPRO-seq: A tool for annotation and profiling of ncRNAs in sRNA-seq data. Bioinformatics.

[15] Benjamini Y., Hochberg Y. (1995). Controlling the False Discovery Rate: A Practical and Powerful Approach to Multiple Testing. J. R. Stat. Soc. Ser. B (Methodol.).

[16] Rohart F., Gautier B., Singh A., Le Cao K.A. (2017). mixOmics: An R package for ‘omics feature selection and multiple data integration. PLoS Comput. Biol..

[17] Cantini L., Isella C., Petti C., Picco G., Chiola S., Ficarra E., Caselle M., Medico E. (2015). MicroRNA-mRNA interactions underlying colorectal cancer molecular subtypes. Nat. Commun..

[18] Wang L., Feng Z., Wang X., Zhang X. (2010). DEGseq: An R package for identifying differentially expressed genes from RNA-seq data. Bioinformatics.

[19] Hsu S.D., Lin F.M., Wu W.Y., Liang C., Huang W.C., Chan W.L., Tsai W.T., Chen G.Z., Lee C.J., Chiu C.M. (2011). miRTarBase: A database curates experimentally validated microRNA-target interactions. Nucleic Acids Res..

[20] Chen Y., Wang X. (2020). miRDB: An online database for prediction of functional microRNA targets. Nucleic Acids Res..

[21] Agarwal V., Bell G.W., Nam J.W., Bartel D.P. (2015). Predicting effective microRNA target sites in mammalian mRNAs. eLife.

[22] Stockel D., Kehl T., Trampert P., Schneider L., Backes C., Ludwig N., Gerasch A., Kaufmann M., Gessler M., Graf N. (2016). Multi-omics enrichment analysis using the GeneTrail2 web service. Bioinformatics.

[23] Subramanian A., Tamayo P., Mootha V.K., Mukherjee S., Ebert B.L., Gillette M.A., Paulovich A., Pomeroy S.L., Golub T.R., Lander E.S. (2005). Gene set enrichment analysis: A knowledge-based approach for interpreting genome-wide expression profiles. Proc. Natl. Acad. Sci. USA.

[24] Cottret L., Wildridge D., Vinson F., Barrett M.P., Charles H., Sagot M.F., Jourdan F. (2010). MetExplore: A web server to link metabolomic experiments and genome-scale metabolic networks. Nucleic Acids Res..

[25] Heinken A., Sahoo S., Fleming R.M., Thiele I. (2013). Systems-level characterization of a host-microbe metabolic symbiosis in the mammalian gut. Gut Microbes.

[26] Gonzalez A.G., Naldi A., Sanchez L., Thieffry D., Chaouiya C. (2006). GINsim: A software suite for the qualitative modelling, simulation and analysis of regulatory networks. Bio Syst..

[27] Yugi K., Kubota H., Hatano A., Kuroda S. (2016). Trans-Omics: How to Reconstruct Biochemical Networks Across Multiple ‘Omic’ Layers. Trends Biotechnol..

[28] Miskov-Zivanov N., Turner M.S., Kane L.P., Morel P.A., Faeder J.R. (2013). The duration of T cell stimulation is a critical determinant of cell fate and plasticity. Sci. Signal..

[29] Kanehisa M., Goto S. (2000). KEGG: Kyoto encyclopedia of genes and genomes. Nucleic Acids Res..

[30] Besse-Patin A., Jeromson S., Levesque-Damphousse P., Secco B., Laplante M., Estall J.L. (2019). PGC1A regulates the IRS1:IRS2 ratio during fasting to influence hepatic metabolism downstream of insulin. Proc. Natl. Acad. Sci. USA.

[31] Tocher D.R., Betancor M.B., Sprague M., Olsen R.E., Napier J.A. (2019). Omega-3 Long-Chain Polyunsaturated Fatty Acids, EPA and DHA: Bridging the Gap between Supply and Demand. Nutrients.

[32] Rodriguez-Cruz M., Serna D.S. (2017). Nutrigenomics of omega-3 fatty acids: Regulators of the master transcription factors. Nutrition.

[33] Pinel A., Rigaudiere J.P., Jouve C., Montaurier C., Jousse C., LHomme M., Morio B., Capel F. (2020). Transgenerational supplementation with eicosapentaenoic acid reduced the metabolic consequences on the whole body and skeletal muscle in mice receiving an obesogenic diet. Eur. J. Nutr..

[34] Rodriguez Melendez R. (2000). Importance of biotin metabolism. Rev. Investig. Clin..

[35] Huergo L.F., Dixon R. (2015). The Emergence of 2-Oxoglutarate as a Master Regulator Metabolite. Microbiol. Mol. Biol. Rev. Mmbr..

[36] Hardwick J.P., Osei-Hyiaman D., Wiland H., Abdelmegeed M.A., Song B.J. (2009). PPAR/RXR Regulation of Fatty Acid Metabolism and Fatty Acid omega-Hydroxylase (CYP4) Isozymes: Implications for Prevention of Lipotoxicity in Fatty Liver Disease. PPAR Res..

[37] Dubois V., Eeckhoute J., Lefebvre P., Staels B. (2017). Distinct but complementary contributions of PPAR isotypes to energy homeostasis. J. Clin. Investig..

[38] Booth A.D., Magnuson A.M., Cox-York K.A., Wei Y., Wang D., Pagliassotti M.J., Foster M.T. (2017). Inhibition of adipose tissue PPARgamma prevents increased adipocyte expansion after lipectomy and exacerbates a glucose-intolerant phenotype. Cell Prolif..

[39] Jones J.R., Barrick C., Kim K.A., Lindner J., Blondeau B., Fujimoto Y., Shiota M., Kesterson R.A., Kahn B.B., Magnuson M.A. (2005). Deletion of PPARgamma in adipose tissues of mice protects against high fat diet-induced obesity and insulin resistance. Proc. Natl. Acad. Sci. USA.

[40] Sethi S., Ziouzenkova O., Ni H., Wagner D.D., Plutzky J., Mayadas T.N. (2002). Oxidized omega-3 fatty acids in fish oil inhibit leukocyte-endothelial interactions through activation of PPAR alpha. Blood.

[41] Zuniga J., Cancino M., Medina F., Varela P., Vargas R., Tapia G., Videla L.A., Fernandez V. (2011). N-3 PUFA supplementation triggers PPAR-alpha activation and PPAR-alpha/NF-kappaB interaction: Anti-inflammatory implications in liver ischemia-reperfusion injury. PLoS ONE.

[42] Puigserver P., Wu Z., Park C.W., Graves R., Wright M., Spiegelman B.M. (1998). A cold-inducible coactivator of nuclear receptors linked to adaptive thermogenesis. Cell.

[43] Yoon J.C., Puigserver P., Chen G., Donovan J., Wu Z., Rhee J., Adelmant G., Stafford J., Kahn C.R., Granner D.K. (2001). Control of hepatic gluconeogenesis through the transcriptional coactivator PGC-1. Nature.

[44] Koo S.H., Satoh H., Herzig S., Lee C.H., Hedrick S., Kulkarni R., Evans R.M., Olefsky J., Montminy M. (2004). PGC-1 promotes insulin resistance in liver through PPAR-alpha-dependent induction of TRB-3. Nat. Med..

[45] Du K., Herzig S., Kulkarni R.N., Montminy M. (2003). TRB3: A tribbles homolog that inhibits Akt/PKB activation by insulin in liver. Science.

[46] Choi C.S., Befroy D.E., Codella R., Kim S., Reznick R.M., Hwang Y.J., Liu Z.X., Lee H.Y., Distefano A., Samuel V.T. (2008). Paradoxical effects of increased expression of PGC-1alpha on muscle mitochondrial function and insulin-stimulated muscle glucose metabolism. Proc. Natl. Acad. Sci. USA.

[47] Gu L., Ding X., Wang Y., Gu M., Zhang J., Yan S., Li N., Song Z., Yin J., Lu L. (2019). Spexin alleviates insulin resistance and inhibits hepatic gluconeogenesis via the FoxO1/PGC-1alpha pathway in high-fat-diet-induced rats and insulin resistant cells. Int. J. Biol. Sci..

[48] White M.F. (2002). IRS proteins and the common path to diabetes. Am. J. Physiol. Endocrinol. Metab..

[49] Honma M., Sawada S., Ueno Y., Murakami K., Yamada T., Gao J., Kodama S., Izumi T., Takahashi K., Tsukita S. (2018). Selective insulin resistance with differential expressions of IRS-1 and IRS-2 in human NAFLD livers. Int. J. Obes..

[50] Nandi A., Kitamura Y., Kahn C.R., Accili D. (2004). Mouse models of insulin resistance. Physiol. Rev..

[51] Valverde A.M., Burks D.J., Fabregat I., Fisher T.L., Carretero J., White M.F., Benito M. (2003). Molecular mechanisms of insulin resistance in IRS-2-deficient hepatocytes. Diabetes.

[52] Zhang J., Ou J., Bashmakov Y., Horton J.D., Brown M.S., Goldstein J.L. (2001). Insulin inhibits transcription of IRS-2 gene in rat liver through an insulin response element (IRE) that resembles IREs of other insulin-repressed genes. Proc. Natl. Acad. Sci. USA.

[53] Ide T., Shimano H., Yahagi N., Matsuzaka T., Nakakuki M., Yamamoto T., Nakagawa Y., Takahashi A., Suzuki H., Sone H. (2004). SREBPs suppress IRS-2-mediated insulin signalling in the liver. Nat. Cell Biol..

[54] Nakagawa Y., Shimano H., Yoshikawa T., Ide T., Tamura M., Furusawa M., Yamamoto T., Inoue N., Matsuzaka T., Takahashi A. (2006). TFE3 transcriptionally activates hepatic IRS-2, participates in insulin signaling and ameliorates diabetes. Nat. Med..

[55] Tao H., Wang M.M., Zhang M., Zhang S.P., Wang C.H., Yuan W.J., Sun T., He L.J., Hu Q.K. (2016). MiR-126 Suppresses the Glucose-Stimulated Proliferation via IRS-2 in INS-1 beta Cells. PLoS ONE.

[56] Maniyadath B., Chattopadhyay T., Verma S., Kumari S., Kulkarni P., Banerjee K., Lazarus A., Kokane S.S., Shetty T., Anamika K. (2019). Loss of Hepatic Oscillatory Fed microRNAs Abrogates Refed Transition and Causes Liver Dysfunctions. Cell Rep..

[57] He L., He X., Lim L.P., de Stanchina E., Xuan Z., Liang Y., Xue W., Zender L., Magnus J., Ridzon D. (2007). A microRNA component of the p53 tumour suppressor network. Nature.

[58] Chakraborty C., Doss C.G., Bandyopadhyay S., Agoramoorthy G. (2014). Influence of miRNA in insulin signaling pathway and insulin resistance: Micro-molecules with a major role in type-2 diabetes. Wiley Interdiscip. Rev. RNA.

[59] Lee J., Padhye A., Sharma A., Song G., Miao J., Mo Y.Y., Wang L., Kemper J.K. (2010). A pathway involving farnesoid X receptor and small heterodimer partner positively regulates hepatic sirtuin 1 levels via microRNA-34a inhibition. J. Biol. Chem..

[60] Cheung O., Puri P., Eicken C., Contos M.J., Mirshahi F., Maher J.W., Kellum J.M., Min H., Luketic V.A., Sanyal A.J. (2008). Nonalcoholic steatohepatitis is associated with altered hepatic MicroRNA expression. Hepatology.

[61] Kong L., Zhu J., Han W., Jiang X., Xu M., Zhao Y., Dong Q., Pang Z., Guan Q., Gao L. (2011). Significance of serum microRNAs in pre-diabetes and newly diagnosed type 2 diabetes: A clinical study. Acta Diabetol..

