# Supplementary material for: An Integrated Analysis of miRNA and Gene Expression Changes in Response to an Obesogenic Diet to Explore the Impact of Transgenerational Supplementation with Omega 3 Fatty Acids
Source: Nutrients. 2020 Dec 17;12(12):3864. doi: 10.3390/nu12123864 (PMC7765958; doi:10.3390/nu12123864)
Supplement: Supplementary file 1 [file nutrients-12-03864-s001.zip › nutrients-1027273-supplementary12.22/supp Table 7.docx]

|  |  | microarray |  |  |  | RT-qPCR |  |
| --- | --- | --- | --- | --- | --- | --- | --- |
| Gene symbol | HFoleic vs Ref | HFepa vs Hfoleic | HFepa vs Ref |  | HFoleic vs Ref | HFepa vs Hfoleic | HFepa vs Ref |
| Agpat6 | 0.64* | 1.14 | 0.74* |  | 0.66 b | 1.17 c | 0.77 b |
| Angptl4 | 0.73* | 1.49 a | 1.09 |  | 0.74 | 1.93 b | 1.42 b |
| Chrebp | 1.47* | 0.93 | 1.36* |  | 1.60 b | 0.66 b | 1.06 |
| Cpt1a | 0.74* | 1.10 | 0.81 a |  | 0.83 c | 0.96 | 0.8 c |
| Dnajb1 | 1.29 | 1.29 a | 1.67* |  | 1.46 b | 1.26 c | 1.84 b |
| Foxo1 | 0.89 | 1.22 a | 1.08 |  | 1.15 | 0.95 | 1.10 |
| Gck | 3.88* | 0.57 a | 2.22 a |  | 3.34 b | 0.68 b | 2.27 b |
| Irs2 | 0.45* | 1.62 a | 0.73 |  | 0.63 b | 1.52 b | 0.95 |
| Hmgcs1 | 2.07* | 0.78 | 1.61 a |  | 2.13 b | 0.78 c | 1.65 b |
| Hspb1 | 1.6* | 1.10 | 1.77* |  | 1.45 | 1.9 c | 2.76 b |
| Gfpt1 | 1.33* | 0.73 a | 0.97 |  | 1.55 b | 0.58 b | 0.90 |
| Pck1 | 0.6* | 1.36 a | 0.82 a |  | 0.5 b | 1.17 | 0.59 b |
| Ppargc1a | 0.69* | 0.95 | 0.65 a |  | 0.96 | 0.88 | 0.84 |
| Sdc1 | 0.59* | 1.16 | 0.69* |  | 0.62 b | 0.63 | 0.39 b |
| Srebf1 | 2.02* | 0.66 a | 1.33 |  | 3.22 b | 0.43 b | 1.39 |
| Xbp1 | 1.60* | 0.70 a | 1.11 |  | 1.77 b | 0.44 b | 0.79 |

**Supplementary Table 7.** RT-qPCR confirmation of microarray expression data. Changes in mRNA levels were determined using microarrays and RT-qPCR. Data are mRNA level ratio between the different groups. *: significant expression change (p < 0.05) with FDR correction identified using microarray expression data; a: significant expression change (p < 0.05) with no FDR correction identified using microarray expression data. Expression change evaluated by RT-qPCR were confirmed at p < 0.05 (b) or p < 0.1 (c).
