# Supplementary figures and images for: An Integrated Analysis of miRNA and Gene Expression Changes in Response to an Obesogenic Diet to Explore the Impact of Transgenerational Supplementation with Omega 3 Fatty Acids
Source: Nutrients. 2020 Dec 17;12(12):3864. doi: 10.3390/nu12123864 (PMC7765958; doi:10.3390/nu12123864)

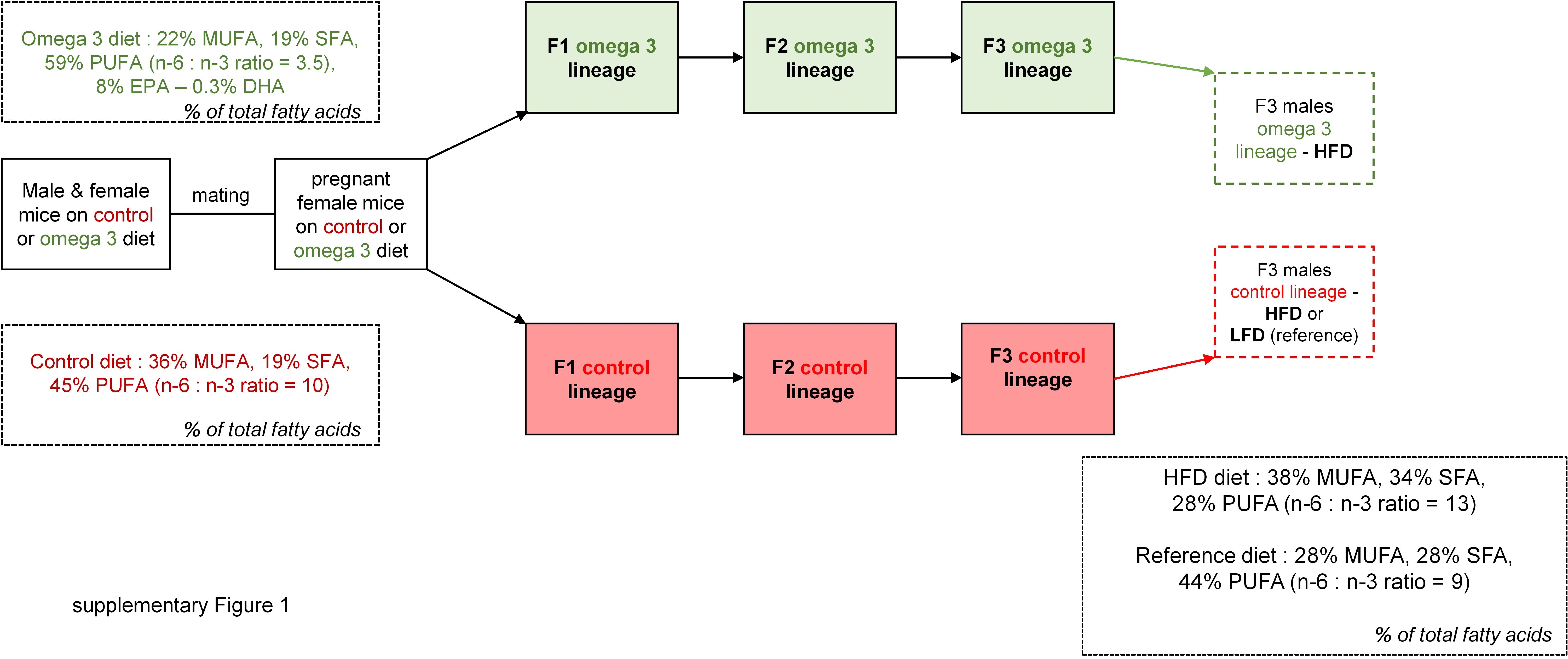

Supplement: Supplementary file 1 [file nutrients-12-03864-s001.zip › nutrients-1027273-supplementary12.22/final sup material R112.22/Suppl F S1.jpg]

→

Link

↔

Reversible link

□

Reaction

○

Metabolites

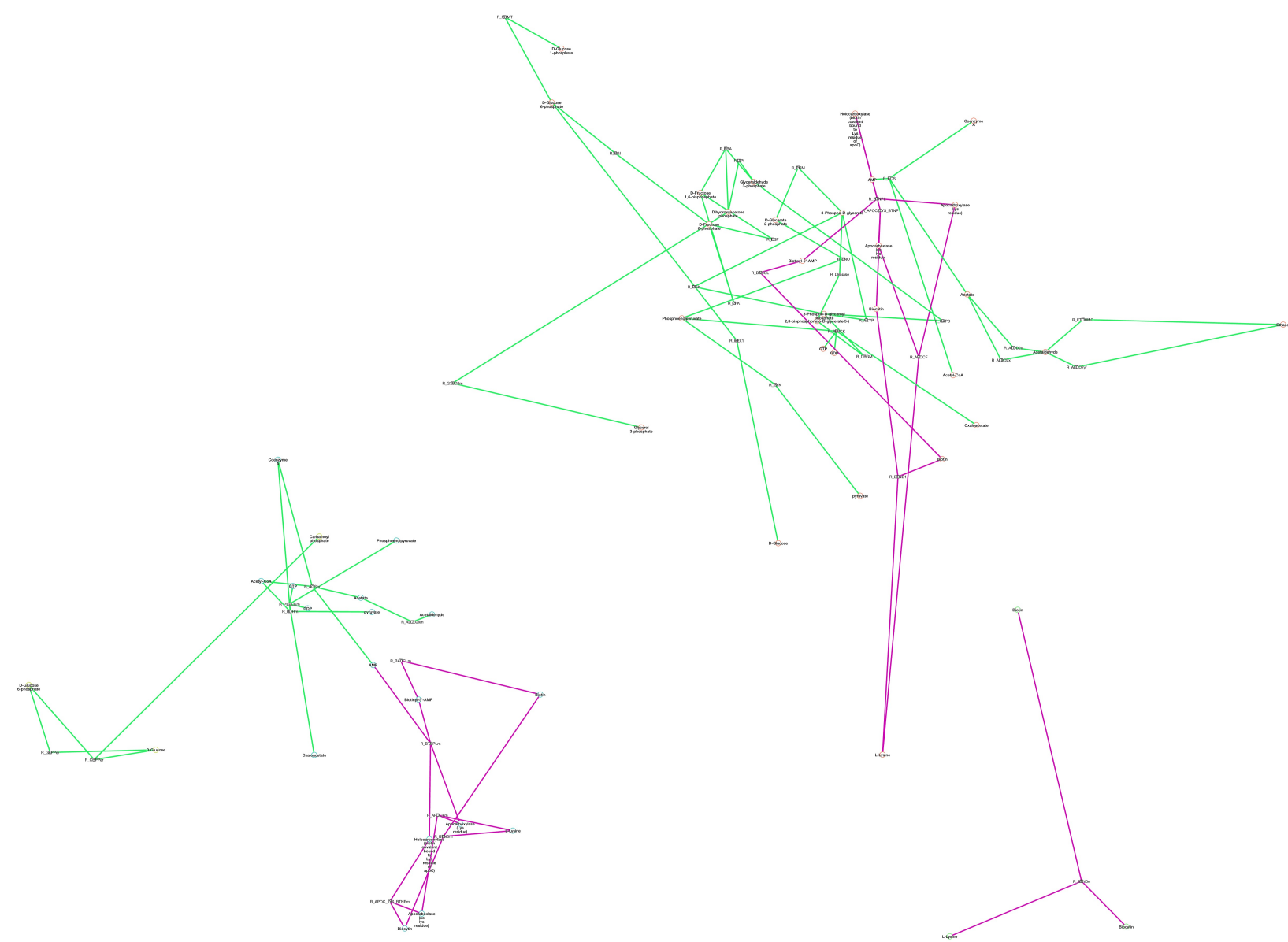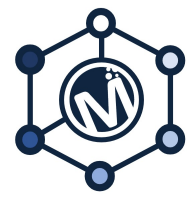

Supplement: Supplementary file 1 [file nutrients-12-03864-s001.zip › nutrients-1027273-supplementary12.22/final sup material R112.22/Suppl F S2A.pdf]

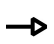 Link

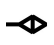 Reversible link

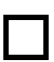 Reaction

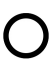 Metabolites

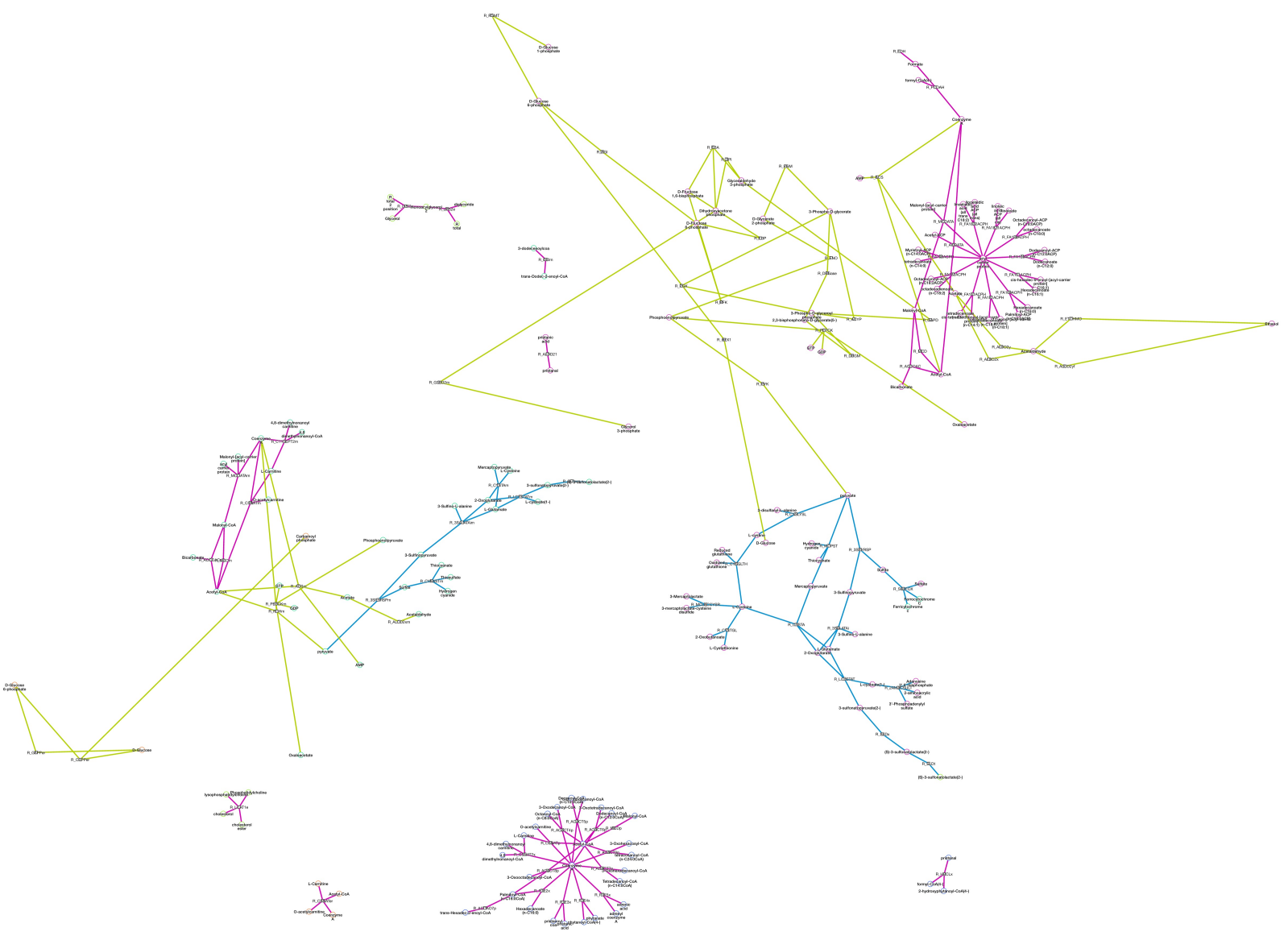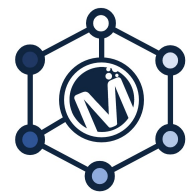

Supplement: Supplementary file 1 [file nutrients-12-03864-s001.zip › nutrients-1027273-supplementary12.22/final sup material R112.22/Suppl F S2B.pdf]
